# Nylon Affinity Networks Capture and Sequester Two Model Bacteria Spiked in Human Plasma

**DOI:** 10.3390/pathogens14080778

**Published:** 2025-08-06

**Authors:** Fatema Hashemi, Silvia Cachaco, Rocio Prisby, Weidong Zhou, Gregory Petruncio, Elsa Ronzier, Remi Veneziano, Barbara Birkaya, Alessandra Luchini, Luisa Gregori

**Affiliations:** 1 Division of Emerging Transfusion-Transmitted Diseases, Office of Blood Research and Review, Food and Drug Administration, Silver Spring, MD 20993, USA; fhashem@gmu.edu (F.H.); scachaco@gmu.edu (S.C.); 2 Center for Applied Proteomics and Molecular Medicine, George Mason University, Manassas, VA 20110, USA; rcornero@gmu.edu (R.P.); wzhou@gmu.edu (W.Z.); bbirkaya@gmu.edu (B.B.); 3 Center for Molecular Engineering, Department of Chemistry & Biochemistry, George Mason University, Manassas, VA 20110, USA; gpetrunc@gmu.edu; 4 Biomedical Research Laboratory, Institute for Biohealth Innovation, George Mason University, Manassas, VA 20110, USA; eronzier@gmu.edu; 5 Department of Bioengineering, College of Engineering and Computing, George Mason University, Manassas, VA 20110, USA; rvenezia@gmu.edu

**Keywords:** pathogen reduction technology, nylon affinity networks, Alcian Blue, transfusion safety, bacterial capture, synthetic dyes

## Abstract

Ensuring bacterial safety of blood transfusions remains a critical focus in medicine. We investigated a novel pathogen reduction technology utilizing nylon functionalized with synthetic dyes (nylon affinity networks) to capture and remove bacteria from plasma. In the initial screening process, we spiked phosphate buffer solution (PBS) and human plasma (1 mL each) with 10 or 100 colony forming units (cfu) of either *Escherichia coli* or *Staphylococcus epidermidis*, exposed the suspensions to affinity networks and assessed the extent of bacterial reduction using agar plate cultures as the assay output. Nineteen synthetic dyes were tested. Among these, Alcian Blue exhibited the best performance with both bacterial strains in both PBS and plasma. Next, bacterial suspensions of approximately 1 and 2 cfu/mL in 10 and 50 mL, respectively, were treated with Alcian Blue affinity networks in three sequential capture steps. This procedure resulted in complete bacterial depletion, as demonstrated by the lack of bacterial growth in the remaining fraction. The viability of the captured bacteria was confirmed by plating the post-treatment affinity networks on agar. Alcian Blue affinity networks captured and sequestered a few plasma proteins identified by liquid chromatography tandem mass spectrometry. These findings support the potential applicability of nylon affinity networks to enhance transfusion safety, although additional investigations are needed.

## 1. Introduction

Blood transfusions are essential medical procedures that contribute significantly to patient survival and recovery. They are particularly crucial in managing trauma, supporting surgical interventions, and facilitating cancer treatment, where they play a key role in restoring blood volume and supporting physiological functions. The National Blood Collection and Utilization Survey highlights the ongoing importance of blood, plasma, and platelet transfusions in the United States healthcare system [1]. In 2021, approximately 10,764,000 units of red blood cells were transfused. Additionally, 2,215,000 units of plasma were administered, while platelet transfusions accounted for 2,175,000 units. These figures underscore the substantial volume of blood components utilized in healthcare and the necessity to maintain a stable and sufficient blood supply.

Despite important advancements in blood safety over the past three decades, particularly the integration of nucleic acid testing to enhance the detection of viral pathogens, bacterial contamination persists as a risk, especially in platelet transfusions [2,3]. In the United States, bacterial contamination ranks as the second leading cause of transfusion-related fatalities [3]. Despite rigorous surveillance efforts, contamination rates for platelets remain concerning, with occurrences ranging from 1 in 2500 to 1 in 5000 units [4]. Bacterial contamination of platelets primarily arises during blood collection and is typically caused by Gram-positive bacteria from normal skin flora or, less frequently, Gram-negative bacteria from asymptomatic donor bacteremia or the environment [4]. Storage of platelets at room temperature with agitation promotes bacterial growth.

In the United States, this risk is mitigated by several strategies described in Food and Drug Administration (FDA) guidance [5], including bacterial culture, rapid testing, and pathogen reduction technologies (PRTs) [2,5,6]. Among the available PRTs, the INTERCEPT system (Amotosalen/UVA) for platelets is the only method approved in the U.S.; other PRTs are the Mirasol system (Riboflavin/UV light, and the THERAFLEX (Macopharma) that uses UV-C light alone [7,8,9]. These PRTs, which primarily function by inhibiting bacterial replication, face limitations such as relatively high upfront costs, lengthy treatment for some methods, limited efficacy against non-enveloped viruses and unintended adverse effects on blood component integrity and function [9,10,11,12]. To date, no PRT has received approval by the FDA to treat whole blood.

We investigated an alternative approach to PRT based on physical removal of bacteria from blood rather than their inactivation. Removal is accomplished by affinity ligands such as dyes that bind bacteria under physiological conditions. We selected nylon as the base material for immobilization of the dyes because of its well-characterized biocompatibility and widespread use in medical applications. Nylon is a versatile synthetic polyamide polymer exhibiting mechanical robustness, flexibility, and chemical resistance [13,14]. Our previous studies showed that synthetic dyes immobilized on biosafe matrices, such as nylon or hydrogels, successfully captured and sequestered low-abundance analytes from plasma and other complex fluids. These dyes bind proteins, carbohydrates, lipids, and nucleic acids through defined affinity constants and mechanisms [15,16,17,18,19,20,21,22]. Building on this foundation, we explored the use of synthetic dyes incorporated into nylon filaments, which were assembled into nonwoven sheets, to capture transfusion-relevant bacteria. We refer to these dye-modified materials as nylon affinity networks. In this study, we evaluated the ability of dyed nylon affinity networks to capture two bacterial strains commonly implicated in blood contamination, *Escherichia coli* and *Staphylococcus epidermidis*, in phosphate buffer saline (PBS) and human plasma. The most promising dye identified in this analysis was tested in sequential capture studies to assess its suitability for further investigations.

## 2. Materials and Methods

### 2.1. Preparation of Nylon Affinity Networks

Nylon 66 sheets (Pellon, Clearwater, FL, USA) obtained from Greatlakes Fibers Company were functionalized with synthetic dyes using both manual and machine-assisted dyeing procedures. Initially, an aliquot of 0.5 g nylon was pre-cleaned in 1% (*w*/*v*) Alconox (Alconox, White Plains, NY, USA) detergent solution for 30 min with gentle agitation, residual detergent was rinsed off in deionized water and the nylon was then dried at 37 °C overnight. Nineteen dyes were tested. The dye bath was prepared by dissolving synthetic dyes in 15 mL of deionized water with adjusted pH and with 0.24 g of sodium chloride. A few dyes also required the addition of 0.24 g sodium 1-naphthalenesulfonate (TCI America, Inc., Portland, OR, USA). Table 1 shows the dyeing conditions for the selected dyes [23]. The cleaned and dried nylon sheets were immersed in the dye solution, with the temperature gradually raised from 25 °C to 100 °C in 1 h. The dye bath was then maintained at 100 °C for an additional hour before being gradually cooled to 25 °C to preserve fiber integrity. Excess dye was removed by rinsing the nylon sheets twice with 50 mL of 0.1% (*w*/*v*) Alconox solution followed by water until the rinse water ran clear. The dyed nylon sheets (nylon affinity networks) were air-dried at 37 °C overnight and stored for further use.

For large-scale preparations, a TD130 IR Lab Dyeing Machine (ATI Corporation, New Holland, PA, USA) was used. Four grams of nylon sheets were cleaned and dried as described above. The dye bath contained 100 mL of deionized water with 3.8 g of sodium chloride adjusted to the required pH (Table 1), and for Alcian Blue, 3.8 g of sodium 1-naphthalenesulfonate was added. The nylon sheets were immersed in the solution, heated from 25 °C to 100 °C in 1 h, maintained at 100 °C for 1 h, and cooled to 25 °C. The sheets were air-dried overnight at 37 °C. Manually and machine-dyed nylon sheets were sterilized by autoclaving in distilled water at 121 °C and 15 psi for 5–10 min and stored semi-wet until use.

### 2.2. Quantitation of Dye Incorporation into Affinity Networks

To quantify dye incorporation into nylon sheets, 6 mg of dyed nylon were dissolved in 1 mL of 22% (*w*/*v*) CaCl_2_/MeOH as the diluent and incubated on a rotator at room temperature for 1 h with periodic vortexing until complete dissolution. A calibration curve for each dye was generated using a 12-point, two-fold dilution series of the stock dye solution (1 mg in 1 mL of diluent) in a 96-well plate, with 150 µL per well in triplicate. In the same plate, dissolved dyed nylon solution was loaded and the absorbance at the dye’s maximum wavelength was measured for all samples using a UV–Vis spectrophotometer (BioTek Cytation™ 5, Agilent, Santa Clara, CA, USA). The calibration curve for each dye was used to estimate the dye incorporated into the nylon sheets. Blanks containing diluent alone were included as controls.

### 2.3. Optimization of pH and Alcian Blue Concentration for Preparation of Nylon Affinity Networks

Optimization of pH in the dyeing step to maximize Alcian Blue incorporation into nylon was measured at 10 pH levels (2–10) maintaining a constant Alcian Blue concentration of 42 mg/mL. Ten nylon sheets of 0.24 g each were dyed at different pHs using the manual protocol. The optimal pH was determined based on fiber integrity and dye incorporation measured as described in Section 2.2.

Once pH 2.5 was identified as optimal, the effect of the dye concentration was assessed. Nylon sheets of 0.24 g were dyed using various amounts of Alcian Blue ranging from 3 mg to 60 mg in 1 mL. The best dye concentration was determined based on dye incorporation measured as in Section 2.2.

### 2.4. Evaluation of Bacterial Bapture in a Single Step

*Escherichia coli* (https://www.atcc.org/products/25922 (accessed on 30 July 2025)) was cultured in Luria–Bertani (LB) broth and *Staphylococcus epidermidis* (https://www.atcc.org/products/12228 (accessed on 30 July 2025)) was cultured in Tryptic Soy broth (TSB) at 37 °C to the logarithmic phase (we used 1 OD_600 nm_ equivalent to 8 × 10^8^ cells/mL for all our stock bacterial estimates) and diluted to approximately 10 cfu/mL and 100 cfu/mL in PBS or a 1:1 mixture of human plasma (Innovative Research, Inc., Novi, MI, USA) in PBS. One milliliter of bacterial suspension was incubated with 45 mg of nylon affinity networks at room temperature for 45 min under gentle agitation. This incubation time was selected to ensure ample time for bacteria binding, but shorter times are expected to be effective as well. Following incubation, the liquid phase containing unbound bacteria was carefully aspirated and two 500 µL aliquots were plated on LB agar. In parallel and as a control, 500 µL of the starting bacterial solution diluted to the targeted 10 cfu and 100 cfu per mL were plated on agar to assess the exact number of bacteria in the spikes. Plates were incubated at 37 °C for 24 h or longer for *S. epidermidis*, and then the number of bacteria in the plates was enumerated. The procedure was repeated using 1:1 human plasma in PBS to evaluate the affinity networks performance under physiologically more relevant conditions.

The percentage of bacteria bound, or percentage depletion, was calculated as 1 − (number of bacteria counted on the plate divided by number of total bacteria in control plate) × 100.

### 2.5. Evaluation of Bacterial Removal in Three Serial Capture Steps

Fifty milliliters of 3.1 ± 0.4 cfu/mL of *E. coli* and 2.1 ± 0.1 cfu/mL of *S. epidermidis* suspensions in PBS were mixed with 90 mg of Alcian Blue affinity networks and incubated at room temperature on a shaker for 45 min. In preliminary studies, incubation of 30 min was sufficient for bacterial binding. The treated suspension containing the unbound bacteria was separated from the nylon and incubated with fresh 90 mg nylon affinity networks. The bacterial capture process was carried out for a total of three sequential steps. After each capture step, 5 mL of the treated suspension was inoculated in duplicate into 45 mL of LB broth to evaluate the presence of bacteria. Following the third step, the remaining suspension was divided into five 5 mL aliquots and then each aliquot was inoculated into 45 mL growth media and allowed to grow for three days. Bacterial concentrations in the spikes were estimated from serial dilutions of the starting stocks plated on agar plates in duplicate.

Optical density at 600 nm was measured on days 1, 2, and 3 in all tubes to monitor bacterial growth (turbidity test). Capture of bacteria by the nylon affinity networks was considered complete if no bacterial growth was observed after day 3. In addition, after each capture step, the nylon networks were placed on agar plates and incubated at 37 °C to allow the captured bacteria to grow.

After validating the sequential capture steps with bacteria suspended in 50 mL of PBS, a sequential filtration method was tested with 10 mL suspensions (*E. coli* and *S. epidermidis*) in either PBS or human plasma at 0.8 ± 0.1 cfu/mL of *E. coli* and 0.7 ± 0.1 cfu/mL of *S. epidermidis* estimated from a serial dilution of bacterial stocks as indicated above. In the first filtration, the bacterial suspension was passed through 90 mg of affinity networks placed in a disposable chromatographic column (1 cm × 4 cm, Bio-Rad laboratories, Hercules, CA, USA). The flow through sample containing unbound bacteria was collected and passed through another column containing fresh 90 mg affinity networks. This step was repeated a third time, always with fresh 90 mg of affinity networks. The flow rate of filtration was controlled to 5 drops per minute. After each filtration, two 1 mL aliquots of the treated suspensions were removed and each inoculated into 9 mL of growth media to assess bacterial growth. Following the final step, the entire flow through was divided into 1 mL aliquots and each aliquot was inoculated into 9 mL media. Bacterial growth in the tubes was followed by measuring the optical density at 600 nm at 1, 2, and 3 days. The affinity networks recovered from the columns were placed on agar plates and bacteria were allowed to grow overnight or longer for *S. epidermidis*.

### 2.6. Plasma Protein Captured by Nylon Affinity Network Analysis

#### 2.6.1. SDS-PAGE and Coomassie Staining

Human plasma (500 µL) was diluted 1:1 with PBS and incubated with 7 mg of Alcian Blue affinity networks at room temperature for 45 min under rotation. The networks were washed twice with 1 mL PBS, and bound proteins were eluted with 50 µL Laemmli buffer. An aliquot of plasma and the unbound and eluted proteins were analyzed by SDS-PAGE on a 4–20% gradient gel in Tris glycine (Novex™, Thermo Fisher Scientific, Waltham, MA, USA) followed by Coomassie staining.

#### 2.6.2. Mass Spectrometry Analysis

Plasma proteins bound to Alcian Blue affinity networks, as described for SDS-PAGE, were eluted with 160 µL of 0.05 µg RapiGest (Waters Corporation) in 50 mM ammonium bicarbonate. To reduce disulfide bonds, 8 µL of 100 mM TCEP (Tris(2-carboxyethyl) phosphine) were added to the mixture, which was then incubated at 90 °C for 5 min. Following reduction, iodoacetamide at a final concentration of 50 mM was introduced to alkylate free cysteine residues. The reaction was allowed to proceed at room temperature in the dark for 20 min. Protein digestion was carried out overnight at 37 °C using sequencing-grade trypsin (Promega Corporation, Madison, WI, USA cat#V5113) at a 1:50 *w*/*w* enzyme-to-substrate ratio in 50 mM ammonium bicarbonate, pH 8. After digestion, the reaction was quenched by adding 2 µL of 100% trifluoracetic acid (TFA). The digested samples were desalted using Pierce C18 spin columns (G-Biosciences, St. Louis, MO, USA) according to the manufacturer’s instructions. Final eluates were dried using a nitrogen evaporator and the samples were reconstituted in 10 µL of 0.1% formic acid for subsequent analysis. LC–MS/MS analysis was performed using a Thermo Scientific Exploris 480 coupled with a nanospray EASY-nLC 1200 UHPLC system. Peptides were separated by reversed-phase chromatography on a PepMap RSLC 75 µm i.d. × 15 cm, 2 µm C18 resin LC column (Thermo Fisher Scientific, Waltham, MA, USA). The mobile phase consisted of 0.1% formic acid in ultrapure water (mobile phase A) and 0.1% formic acid, 80% acetonitrile (mobile phase B). A linear gradient was applied, starting from 5% mobile phase B to 50% mobile phase B over 90 min, followed by an increase to 100% mobile phase B for an additional 2 min at a flow rate of 300 nL/min.

The Exploris 480 mass spectrometer was operated in data-dependent mode, with each full MS scan followed by TopN MS/MS scans of the most abundant peptide ions (charge states 2+ to 4+). These ions were dynamically selected for collision-induced dissociation (CID) with a normalized collision energy of 35%. Tandem mass spectra were acquired and searched against the NCBI human database using Proteome Discoverer 2.1 software using tryptic cleavage constraints.

## 3. Results

### 3.1. Alcian Blue Is the Best Binder of E. coli Among All Dyes Tested

**Figure 1:**
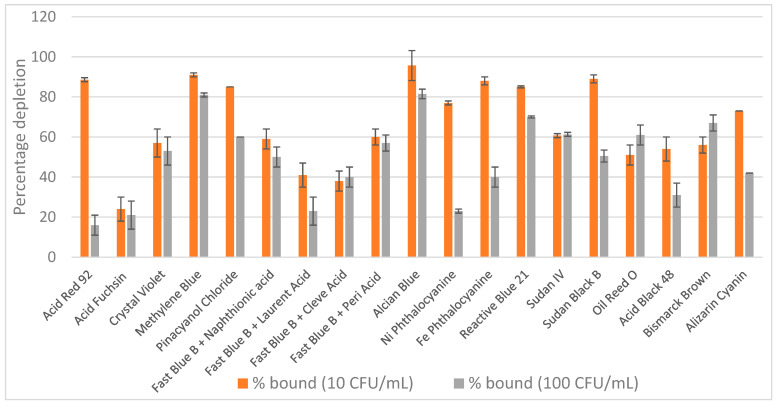
Screening of 19 dyed nylon affinity networks for their ability to capture *E. coli* spiked in PBS. The *y*-axis represents the percentage of bacteria depleted by each dyed nylon material calculated based on the starting bacterial loads. The error bars represent the standard deviation calculated from at least two independent experiments run with duplicate plates.

Nineteen dyes, including Acid Red 92, Acid Fuchsin, Crystal Violet, Methylene Blue, Pinacyanol Chloride, Fast Blue B (reacted with naphthionic, laurent, cleve, or peri acids), Alcian Blue Pyridine variant, Ni Phthalocyanine, Fe Phthalocyanine, Reactive Blue 21, Sudan IV, Sudan Black B, Oil Red O, Acid Black 48, Bismarck Brown Y, and Alizarin Cyanin, were selected to span a wide range of chemical classes and binding properties. These included acid and basic dyes, diazonium salts, hydrophobic and planar aromatic compounds, metal complexes, azo dyes, and anthraquinone derivatives. Each dye was incorporated into nylon sheets and screened for its ability to bind an *E. coli* suspension in PBS with two bacterial concentrations (10 cfu/mL and 100 cfu/mL) using a 1 mL assay format with single-step capture. The results of this initial screen are shown in Figure 1. Dyes exhibiting greater than 80% bacterial binding for least one bacterial concentration were selected for further investigation. Seven dyes met this criterion: Methylene Blue, Pinacyanol Chloride, Alcian Blue, Reactive Blue 21, Acid Red 92, Fe Phthalocyanine and Sudan Black B. However, only two dyes were selected: Alcian Blue and Reactive Blue 21 and all others had to be excluded from subsequent experiments due to either commercial discontinuation or other technical reasons.

The remaining two best dyes were then tested for their ability to capture *Staphylococcus epidermidis* in PBS at two concentrations. Table 2 presents results from at least three independent experiments with *E. coli* and *S. epidermidis* spiked in PBS at both 10 and 100 cfu/mL. Alcian Blue was selected as the best performer among all dyes tested across all experimental conditions and was used in all successive tests.

### 3.2. Optimization of Alcian Blue Incorporation into Nylon

**Figure 2:**
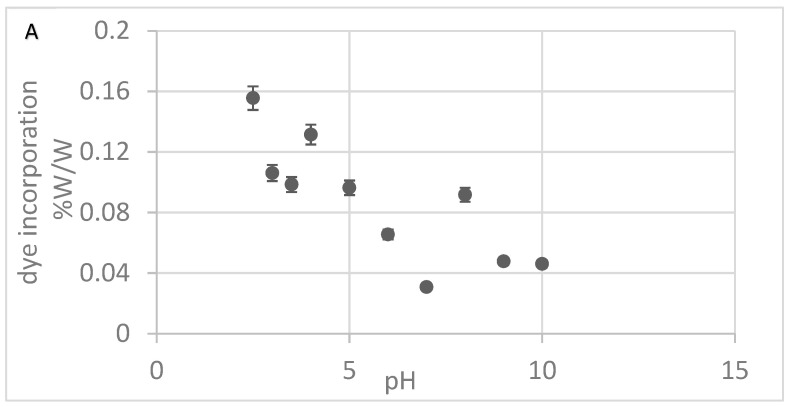

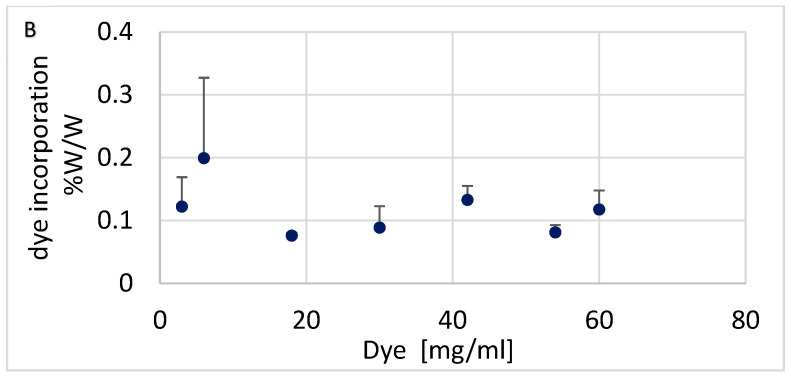
(**A**) Alcian Blue incorporation at different pHs. (**B**) Alcian Blue incorporation at different dye concentrations. The error bars represent the standard deviation calculated from three replicates.

Efforts to optimize Alcian Blue incorporation into nylon focused on two key parameters: pH and the dye concentration in the dyeing solution. As shown in Figure 2A, dye incorporation decreased with increasing pH, with the highest incorporation observed between pH 2.5 and 3.0, suggesting that strong electrostatic interactions under acidic conditions facilitate dye binding to the nylon matrix. Using the optimal pH of 2.5, we varied the dye concentration from 3 mg to 60 mg/mL and measured the dye incorporated into the nylon. Dye incorporation exhibited minor variations across the tested concentration range, with no clear dose-dependent effect (Figure 2B). Based on these results, a working concentration range of 32–42 mg/mL was selected to ensure excess dye was available to color the nylon. These optimized conditions of pH and dye concentration were applied to produce Alcian Blue nylon affinity networks using water-based chemistry and machine-assisted fabrication for all studies.

**Figure 3:**
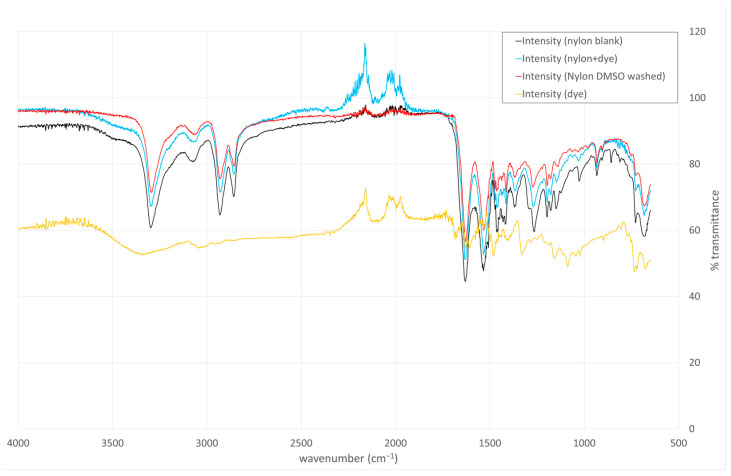
Organic solvent washes and FT-IR analysis support the hypothesis that the dye–nylon association is mediated by hydrophobic interactions. Undyed nylon: black, Alcian Blue dyed nylon: Blue, DMSO-washed dyed nylon: red, Alcian Blue dye: orange.

To probe the nature of the dye–nylon interaction, we subjected the dyed nylon to washes with an alkaline aqueous solution (pH 9) or with dimethyl sulfoxide (DMSO), which are expected to disrupt ionic and hydrophobic interactions, respectively. The dye remained bound after the alkaline wash but was removed upon treatment with DMSO. In the FT-IR spectrum, we monitored the region above 2000 cm^−1^, where a peak consistent with high-energy aromatic C–H bending, present in both the free Alcian Blue dye and the dyed nylon, was no longer observed following DMSO washing (Figure 3). This pattern supports the conclusion that hydrophobic interactions play a dominant role in mediating the dye–nylon association.

### 3.3. Scanning Electron Microscopy Reveals That Alcian Blue Enhances Bacterial Binding to Nylon

**Figure 4:**
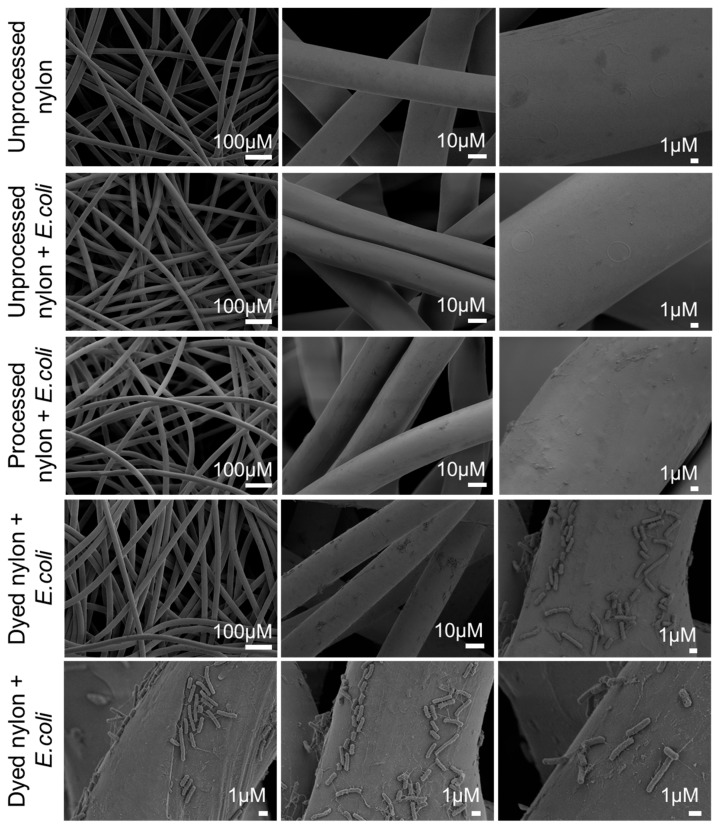
Scanning electron microscopy demonstrates that incorporation of Alcian Blue into the nylon matrix enables the capture of *E. coli* from suspension. Top row: unprocessed nylon imaged before *E. coli* exposure shows smooth fiber surfaces. Second row: unprocessed nylon incubated with *E. coli* in PBS shows no evidence of bacterial binding. Third row: processed nylon (exposed to the dye bath without dye) displays increased surface irregularities but no bacterial adhesion. Bottom two rows: Alcian Blue nylon incubated with *E. coli* shows significant bacterial capture and adhesion to the nylon.

Scanning electron microscopy (SEM) was used to visualize nylon fibers at various stages of the dyeing process and to assess whether Alcian Blue enhances the ability of nylon to bind bacteria. SEM images were obtained for the following conditions: (1) unprocessed nylon, (2) unprocessed nylon exposed to *E. coli*, (3) nylon exposed to the dye bath without dye (processed nylon) subsequently exposed to *E. coli*, and (4) Alcian Blue nylon exposed to *E. coli*. Materials in conditions 2–4 were exposed to *E. coli* for 60 min (Figure 4). The SEM images show that unprocessed nylon has a smooth and regular surface, and no bacterial adhesion was observed following exposure to *E. coli*. Processed nylon exhibited increased surface defects compared to unprocessed nylon, likely due to exposure to acidic and salt-rich conditions. However, these changes in surface topography resulted in no visible bacterial capture. In contrast, Alcian Blue nylon showed clear evidence of rod-shaped bacteria firmly attached to the nylon surface, including clusters and possibly early microcolonies. These results indicate that dye incorporation enables bacterial capture.

### 3.4. Alcian Blue Nylon Networks Captured Bacteria Spiked in 1 mL of PBS or Human Plasma

**Figure 5:**
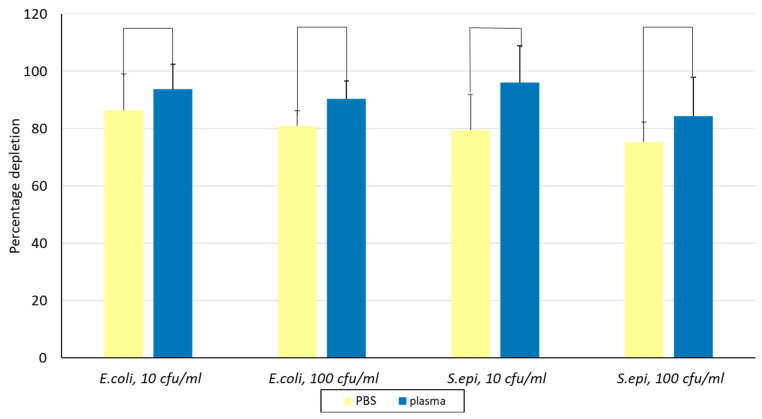
Evaluation of depletion of *E. coli* and *S. epidermidis* in PBS and 1:1 human plasma at two concentrations using Alcian Blue affinity networks. Binding was conducted in 1 mL suspensions and 0.5 mL was plated in duplicate on agar. The error bars represent the standard deviation calculated from at least two independent experiments run on duplicate plates. *S. epi*., *S. epidermidis*.

Aliquots of 45 mg of Alcian Blue affinity networks were incubated with *E. coli* and *S. epidermidis* at concentrations of 10 cfu/mL and 100 cfu/mL in a 1 mL assay format using either PBS or a 1:1 mixture of PBS and human plasma. The results shown in Figure 5 are reported as PBS versus plasma for all conditions tested. The percentage depletion for *E. coli* and *S. epidermidis* was >80% and >75%, respectively. No statistically significant differences were observed between plasma and PBS or between the two bacterial concentrations for the same bacterial strain, suggesting that plasma proteins did not interfere with bacteria binding (t test *p* values 0.24, 0.07, 0.1, and 0.42). These results confirmed robust bacterial capture across all tested conditions.

### 3.5. Alcian Blue Affinity Networks Captured Bacteria in 50 mL of PBS Using Sequential Batch Incubations

**Figure 6:**
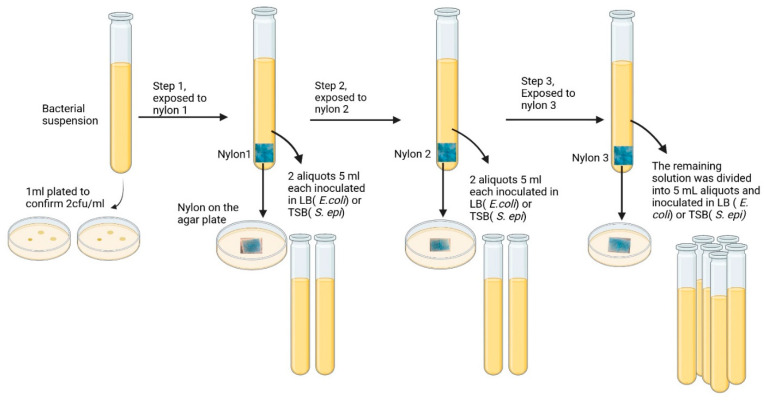
Schematic of sequential batch incubation of 50 mL bacterial suspensions (2 cfu/mL) with three nylon affinity networks (90 mg each). After each exposure, two 5 mL aliquots were transferred to bacterial growth media (LB for *E. coli* or TSB for *S. epidermidis*), and the nylon sheet was plated on agar. The procedure was repeated for three sequential incubations.

To assess the performance of Alcian Blue affinity networks in larger sample volumes, we tested their ability to capture bacteria spiked in 50 mL of PBS using a three-step serial batch incubation protocol. Figure 6 shows a graphic representation of the main steps. Each incubation used 90 mg of dyed nylon to ensure excess surface area for bacterial capture. The initial bacterial suspension containing ~2 cfu/mL, as confirmed by plating 1 mL aliquots of suspension on agar plates, was mixed with the nylon affinity networks. At each step, the unbound bacterial suspension was incubated with a fresh nylon network. Following each incubation, two 5 mL aliquots were transferred into growth media (LB for *E. coli*, TSB for *S. epidermidis*), and bacterial proliferation was monitored over three days via OD_600_ measurements.

After the third incubation step, the remaining suspension was transferred to growth media and monitored for bacterial growth. To confirm bacterial capture, each nylon sheet after exposure to bacteria was plated on agar. Figure 5 shows the bacterial growth patterns over 3 days. After the first capture step, bacteria grew in media as demonstrated by the increase of OD_600_ over three days of incubation. In all cases, duplicated aliquots showed a maximal optical density at 600 nm of 2. A similar trend was observed after the second step. However, no growth was detected after the third capture step in all tubes, indicating complete removal of viable bacteria. The detection limit of the bacterial culture was 1 cfu/mL established as the minimum bacterial concentration yielding OD_600_ > 0 in 3/3 culture replicates.

**Figure 7:**
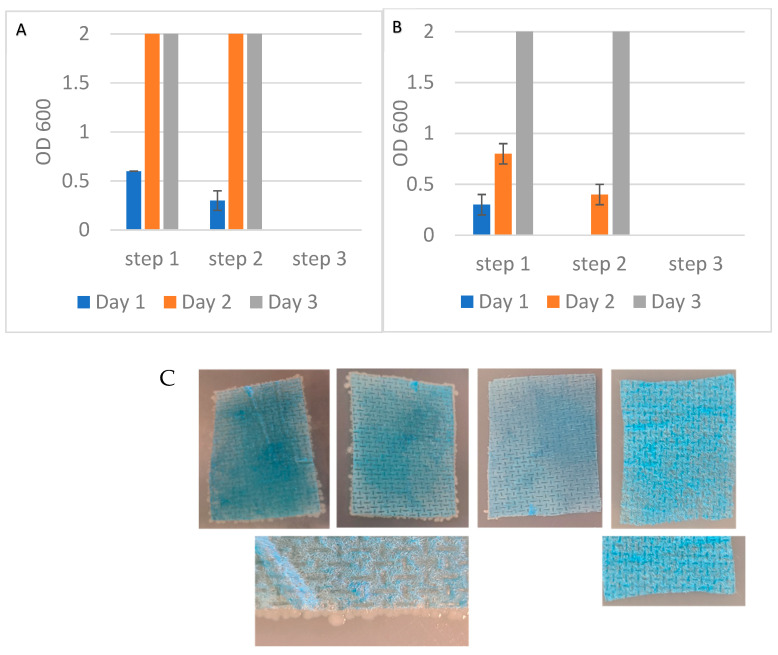
(**A**) Optical density at 600 nm of *E. coli* recovered in the unbound fractions across three sequential exposure steps to nylon affinity networks grown in LB media. (**B**) OD_600_ measurements of *S. epidermidis* recovered in the unbound fractions as in (**A**). (**C**) Affinity networks placed on agar plates after exposure to *E. coli* in three sequential steps: nylon 1 (left), nylon 2 (center), nylon 3 (right) and control unexposed nylon (far right). The figures’ closeups are nylon 1 and unexposed nylon. The size of each nylon affinity networks was approximately 3.2 cm × 4.5 cm. The error bars represent the standard deviation calculated from at least two independent experiments run on duplicate plates.

These results were consistent across both *E. coli* and *S. epidermidis* (Figure 7A,B). Plating the exposed nylon networks on agar further confirmed bacterial capture with visible colonies forming around the edges and on top of the nylon. Unexposed nylon is shown as a control (Figure 7C). These findings demonstrated that Alcian Blue nylon affinity networks removed low levels of bacteria from larger sample volumes.

### 3.6. Alcian Blue Affinity Networks Captured Bacteria in 10 mL Volumes Using Sequential Flow Through Incubations

**Figure 8:**
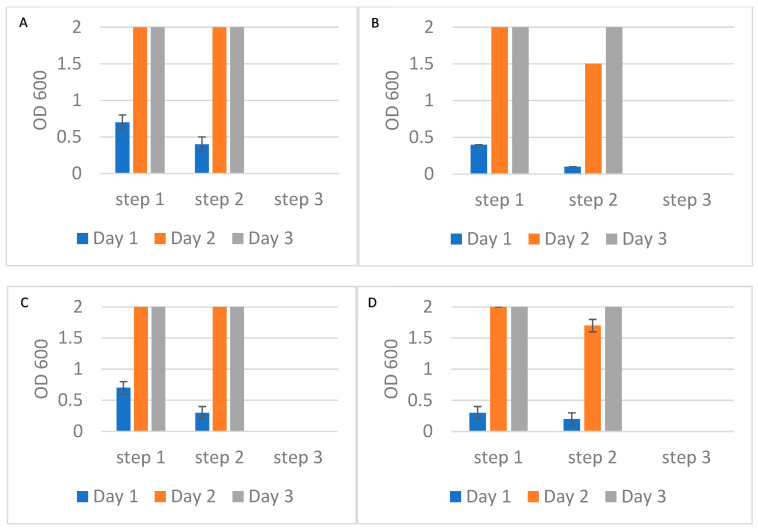
Optical density at 600 nm of bacterial growth in the unbound fractions collected after each of three sequential filtrations using Alcian Blue nylon affinity networks. (**A**) *E. coli* spiked in PBS. (**B**) *S. epidermidis* spiked in PBS. (**C**) *E. coli* spiked in human plasma. (**D**) *S. epidermidis* spiked in human plasma. The error bars represent the standard deviation calculated from at least two independent experiments run on duplicate plates.

To further evaluate the potential of Alcian Blue affinity networks for practical applications, we tested a flow-through assay format where the affinity networks were placed into a chromatographic column and bacteria were suspended in the mobile phase. This configuration mimicked a potential clinical application where the networks could be integrated into blood treatment cartridges or filtration devices. In this experiment, 10 mL suspensions of *E. coli* and *S. epidermidis* at ~1 cfu/mL were passed through three consecutive columns, each containing 90 mg of dyed nylon affinity networks. Experiments were conducted using either PBS or human plasma as the suspension medium. A flow rate of approximately 0.25 mL/min was maintained, providing a total contact time of 40 min per 10 mL sample. This flow rate was chosen to achieve an incubation time similar to that used in previous experiments. Faster flow rates will be evaluated in the future. At each filtration step, two 1 mL aliquots of the unbound fraction were collected and incubated in growth media. Bacterial growth was monitored by measuring the optical density at 600 nm on days 1, 2, and 3 (Figure 8). After the third filtration, the remaining suspensions were incubated with growth media to assess residual bacteria. In all tested conditions, bacterial growth was observed after the first and second filtration steps but was completely absent following the third step, indicating effective and complete removal of bacteria by the affinity networks (Figure 8). These results were consistent for both *E. coli* and *S. epidermidis* in PBS and plasma. Importantly, the presence of plasma proteins did not hinder bacterial capture, confirming the networks’ performance in complex biological fluids.

### 3.7. Alcian Blue Affinity Networks Capture Low Amounts of Human Plasma Proteins

**Figure 9:**
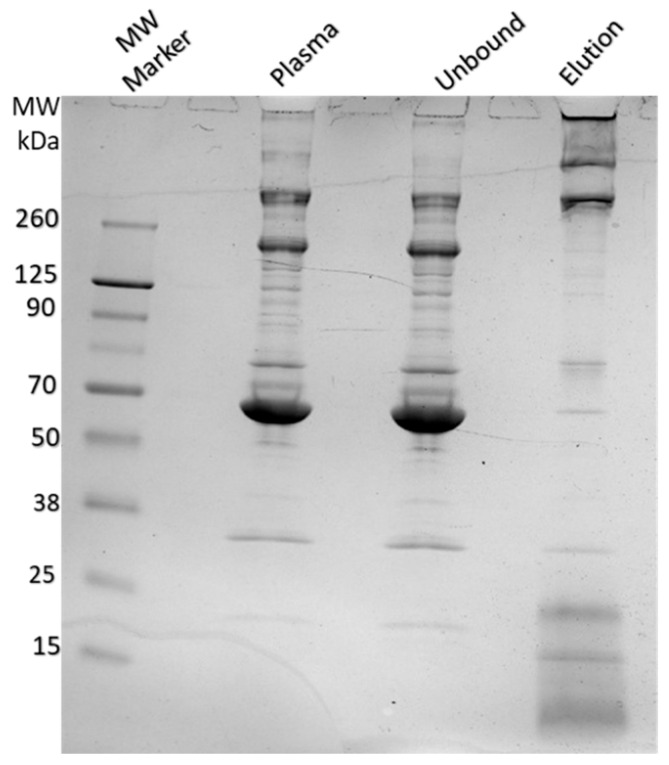
SDS-PAGE analysis of human plasma, unbound, and eluted protein fractions after incubation with Alcian Blue nylon affinity networks. The lanes from left to right show the molecular weight marker, human plasma, unbound fraction, and protein eluted from the affinity networks.

To evaluate the extent of non-specific protein binding, we analyzed plasma proteins retained by the Alcian Blue nylon affinity networks using SDS-PAGE and mass spectrometry. Figure 9 shows a representative SDS-PAGE gel with lanes corresponding to total plasma, the unbound fraction, and proteins eluted from the nylon networks. The affinity network was incubated with 500 µL of human plasma, and the eluted proteins were analyzed by SDS-PAGE. For comparison, 1 µL of plasma and 1 µL of unbound fraction were also loaded onto the gel. The gel revealed that small amounts of proteins, mostly with molecular weights higher than 260 KDa, were captured by the affinity networks. These high molecular weight proteins were fibrinogen, fibronectin and apolipoprotein B 100 subunits as assessed by mass spectrometry of the gel-extracted protein bands. No visible coagulation of plasma was observed following incubation of the affinity networks with plasma.

Label free bottom-up discovery tandem mass spectrometry was employed to determine the proportion of plasma proteins captured by the nylon affinity networks. The results of this analysis are reported in Supplementary Materials (Table S1). The nylon affinity networks captured 0.08% of plasma proteins measured using the peptide spectrum matches (PSMs) as a measure of peptide mass and the formula (PSMs in nylon)/(PSMs in plasma) × 100 (Table S1). The captured proteins primarily included apolipoproteins, fibronectin, and fibrinogen isoforms. These findings confirmed the results from the extracted protein bands discussed above.

## 4. Discussion

This study highlights the promising potential of nylon affinity networks functionalized with Alcian Blue as a novel approach for bacterial reduction in human plasma, with future applications aimed at treating platelet units. Although not explored in this work, it would be interesting to investigate the possibility of incorporating the affinity networks into component manufacturing steps; for example, affinity nylon networks could be coupled with leukoreduction filters to provide removal of leukocytes and bacteria in one step, or they could be used following another PRT method to achieve higher levels of bacterial reduction.

Plasma was selected as a test matrix due to its high protein content, which poses a significant challenge for non-specific interactions, and because platelets are typically resuspended in plasma for storage and transfusion. Unlike conventional pathogen reduction technologies that rely on bacterial inactivation, this strategy is based on physical removal of bacteria. This is conceptually analogous to the use of leukocyte reduction filters to remove white blood cells from blood products [24].

In this work, we selected two transfusion transmission-relevant bacterial species, *E. coli* and *S. epidermidis* (as Gram-negative and Gram-positive organisms, respectively). By evaluating both a Gram-negative and a Gram-positive organism, we aimed to assess the broader applicability of our affinity network approach across diverse bacterial classes.

Additional species will be evaluated in future studies. If further validated, this approach may offer potential advantages over current PRTs. Specifically, capturing and removing bacteria might cause fewer adverse effects on blood components compared to conventional inactivation methods that rely on UV light and chemical additives, which in some cases require quenching or removal of the by-products, with associated costs. The challenge of removal of bacteria is the need to effectively deplete bacteria across a range of species and concentrations while avoiding unintended effects such as platelet activation or aggregation following exposure to the dye and the affinity networks. It should be noted that two common drawbacks of all PRTs, including ours, compared to bacterial detection methods are that the contaminated units are not flagged and thus, this information cannot be used for active surveillance or look-back investigations, and the bacterium contaminating the unit is not identified.

Previous research supports the feasibility of using dye-functionalized nylon for adsorptive separation of biomolecules [13,25]. A key limitation of small-molecule probes is the potential for non-specific binding, as their interactions are often driven by general structural or physicochemical features (e.g., hydrophobicity, charge) rather than unique epitopes. However, in our case, few plasma proteins were found bound to Alcian Blue affinity networks, mostly clustered in the coagulation pathways, apolipoproteins, fibrinogen, and others. SDS-PAGE confirmed that low amounts of these proteins were removed from plasma. Interestingly, high abundance proteins such as albumin, ferritin and immunoglobulin were not among the captured proteins confirming low levels of non-specific binding. No sign of coagulation was observed in the plasma exposed to the affinity networks. Although these results are encouraging, further studies are needed to assess whether the networks may impact the final products’ integrity and physiological functions such as platelet activation and aggregation or triggering coagulation processes.

Screening of multiple small dye molecules led to the selection of Alcian Blue as the best dye. Alcian Blue is a cationic molecule with established biomedical applications, such as glycosaminoglycan, and polysaccharide staining in *E. coli* and *Klebsiella* [26] and for sentinel lymph node visualization in cancer patients [27]. It also stains biofilm-associated acid polysaccharides from *E. coli* and *Listeria monocytogenes* [28] and biofilms from *S. epidermidis* [29]. Hydrophobic interactions are expected to play a dominant role in mediating the dye–nylon association. This is supported by the dyeing conditions, which involve a salt-rich, acidic aqueous bath (pH 2.5, containing 2% NaCl and sodium 1-naphthalenesulfonate), known to favor hydrophobic interactions. Additional evidence comes from post-dyeing washes: the dye remained bound after treatment with alkaline water (pH 9) but was removed upon exposure to dimethyl sulfoxide (DMSO), a solvent that effectively disrupts hydrophobic interactions (Figure 3). Dye incorporation was optimized using water-based chemistries and was approximately 0.12% *w*/*w* (dye weight/nylon weight). This ratio is within the acceptable range for this type of reaction [23]. The interaction between the affinity networks and bacterial cells is likely driven by electrostatic or hydrogen bonding interactions between Alcian Blue and negatively charged surface structures such as glycosaminoglycans and polysaccharides. These interactions appear to be conserved across different bacterial species, suggesting broad-spectrum potential. Notably, the captured bacteria remained viable and could proliferate while attached to the nylon, which may be advantageous in reducing the likelihood of bacterial endotoxin release during capture.

Two independent experiments using sequential capture steps confirmed the robustness of bacterial removal. One experiment with 50 mL of approximately 2 cfu/mL was conducted batch-wise and the second experiment with 10 mL of 1 cfu/mL was performed in flow through mode. Both experimental processes required three capture steps to eliminate all detectable bacteria, regardless of the initial bacterial load. This suggests that saturation of binding sites was not a limiting factor. Based on the measured dye incorporation (0.12% *w*/*w*), 90 mg of nylon corresponded to approximately 83 nmol of Alcian Blue per step. Based on published estimates, a single *E. coli* cell may express approximately 10^6^ to 10^7^ surface-associated polysaccharide structures, including lipopolysaccharides, capsular polysaccharides, outer membrane associated proteins, and components of the peptidoglycan layer [30]. Given that 83 nmol of Alcian Blue corresponds to ~5 × 10^16^ dye molecules, the number of available dye molecules per bacterial cell in our assay exceeds potential binding sites by several orders of magnitude even if we assume that each bacterium engages with multiple dye molecules in the binding. These data suggest that binding efficiency is probably limited by accessibility and surface presentation of the dye rather than by stoichiometric availability. Thus, optimizing dye orientation and surface presentation could offer an important avenue for increasing capture efficiency. To improve performance, strategies such as introducing molecular spacers or crosslinkers between the nylon and dye could enhance accessibility of bacterial targets. Future work will explore these options as well as incorporation into hydrogel or layered formats.

Bacterial contamination levels in platelet units could range from <1 cfu/mL at the time of collection to >10^8^ cfu/mL after several days of storage [31]. While diagnostic testing is typically delayed, allowing bacteria to growth, removal strategies would benefit from immediate application post-collection to prevent bacterial proliferation, which could overwhelm the capture method. Our data showed 2 log_10_ of bacterial reduction, the maximum demonstrable with the used experimental design.

In conclusion, we present evidence that a nylon affinity network functionalized with Alcian Blue can effectively capture two transfusion-relevant bacteria in human plasma with minimal non-specific protein binding. This establishes the proof-of concept for an alternative pathogen reduction method that could be used to improve blood transfusion safety.

## Figures and Tables

**Table 1:** Dye bath conditions and λmax for promising dyes.

Dye	CAS Number	Molecular Weight	Amount of Dye (g/mL)	pH	Dispersing Agent	Sodium Chloride (g/mL)	λmax (nm)
Reactive Blue 21, C_40_H_25_CuN_9_O_14_S_5_	12236-86-1	1079.55	0.12	8	none	0.02	660
Pynacyanol Chloride C_25_H_25_ClN_2_	2768-90-3	388.93	0.09	5.5	none	0.02	600
Methylene Blue C_16_H_18_ClN_3_S · xH_2_O	122965-43-9	319.85 (anhydrous basis)	0.09	10	none	0.02	667
Alcian Blue C_56_H_40_Cl_4_CuN_12_	123439-83-8	1086.36	0.07	2.5–3	Sodium 1-naphthalenesulfonate CAS: 130-14-3	0.02	610
Sudan IV C_24_H_20_N_4_O	85-83-6	380.44	0.09	5.5	none	none	520

**Table 2:** Percentage depletion of *E. coli* and *S. epidermidis* spiked in PBS captured by dyed nylon affinity networks.

Nylon with Various Dyes	*E. coli*		*S. epidermidis*	
	10 cfu/mL	100 cfu/mL	10 cfu/mL	100 cfu/mL
Alcian Blue	95 (±0.7) *	80 (±0.7)	75 (±1.4)	78 (±1.4)
Reactive Blue 21	88 (±1.4)	66 (±0.7)	69 (±0)	68 (±1.4)

* In parenthesis are the standard deviations calculated on at least two independent experiments run on duplicate agar plates.

## Data Availability

All generated mass spectrometry raw data will be deposited in ProteomeXchange upon manuscript acceptance.

## Supplementary Materials

The following supporting information can be downloaded at: https://www.mdpi.com/article/10.3390/pathogens14080778/s1. Table S1. Summary mass spectroscopy analysis of human plasma proteins bound to Alcian Blue nylon affinity networks. Complete results of mass spectroscopy analysis of human plasma proteins and proteins bound to Alcian Blue affinity networks and eluted from the nylon are also included in two Excel spreadsheets: Hashemi et al. Plasma_Peptide and Hashemi et al. Elut_Peptide.
