# Supplementary material for: Nylon Affinity Networks Capture and Sequester Two Model Bacteria Spiked in Human Plasma
Source: Pathogens. 2025 Aug 6;14(8):778. doi: 10.3390/pathogens14080778 (PMC12389278; doi:10.3390/pathogens14080778)
Supplement: Supplementary file 1 [file pathogens-14-00778-s001.zip › Table S1.pdf]

Table S1. Summary mass spectroscopy analysis of human plasma proteins bound to alcian blue nylon affinity networks.

Protein Group ID	# Proteins	# Unique Peptides	# Peptides	# PSMs	Group Description
29	2	27	27	31	apolipoprotein B-100 precursor
21	2	16	16	29	fibrinogen gamma chain isoform gamma-B precursor
23	2	15	15	37	apolipoprotein A-I isoform 1 preproprotein
13	2	13	13	31	fibrinogen alpha chain isoform alpha-E preproprotein
17	2	11	11	30	fibrinogen beta chain isoform 1 preproprotein
5	1	8	8	10	serum albumin preproprotein
10	2	8	8	10	apolipoprotein E isoform a precursor
6	1	7	7	21	vitronectin precursor
20	2	7	7	13	prothrombin isoform 1 preproprotein
7	34	7	7	11	fibronectin isoform X1
22	2	5	5	7	clusterin isoform X1
3	1	4	4	14	apolipoprotein C-II precursor
11	1	2	2	15	apolipoprotein C-III precursor
24	2	2	2	4	hyaluronan-binding protein 2 isoform 1 preproprotein
1	1	2	2	4	inter-alpha-trypsin inhibitor heavy chain H2 precursor
30	5	2	2	2	complement C4-B preproprotein
15	3	2	2	2	coagulation factor IX isoform 1 preproprotein
4	6	1	1	3	src substrate cortactin isoform c
9	1	1	1	2	unconventional myosin-XV isoform X3
27	1	1	1	2	transthyretin precursor
32	3	1	1	1	rho GTPase-activating protein 29 isoform a
31	1	1	1	1	Fanconi anemia group B protein
2	1	1	1	1	secreted phosphoprotein 24 isoform X1
28	1	1	1	1	apolipoprotein A-IV precursor
25	2	1	1	1	inter-alpha-trypsin inhibitor heavy chain H1 isoform a preproprotein
16	4	1	1	1	SAA2-SAA2 protein precursor
8	2	1	1	1	coagulation factor X isoform 1 preproprotein
14	3	1	1	1	inter-alpha-trypsin inhibitor heavy chain H3 isoform X1
19	2	1	1	1	vitamin K-dependent protein S isoform 1 precursor
18	3	1	1	1	uncharacterized protein LOC643802 isoform X1
26	1	1	1	1	microtubule-associated tumor suppressor 1 isoform 4
33	1	1	1	1	protein SOGA3 precursor
